# Protective effects of Silibinin and cinnamic acid against paraquat-induced lung toxicity in rats: impact on oxidative stress, PI3K/AKT pathway, and miR-193a signaling

**DOI:** 10.1007/s00210-024-03511-y

**Published:** 2024-10-25

**Authors:** Basma M. Fouad, A. A. Abdel-Ghany, Mohamed A. Kandeil, Ibrahim T. Ibrahim

**Affiliations:** 1 Department of Biochemistry, Faculty of Pharmacy, Nahda University, Beni-Suef, 62513 Egypt; 2 Biochemistry Department, Faculty of Pharmacy, Al-Azher University, Assiut, Egypt; 3 Department of Biochemistry, Faculty of Veterinary Medicine, Beni-Suef University, Beni-Suef, 62511 Egypt; 4 Department of Biochemistry, Faculty of Pharmacy, Beni-Suef University, Beni-Suef, 62514 Egypt

**Keywords:** Paraquat, Silibinin, Cinnamic acid, MiRNA 193a, PI3K, AKT

## Abstract

Levels of reactive oxygen species (ROS) are the primary determinants of pulmonary fibrosis. It was discovered that antioxidants can ameliorate pulmonary fibrosis caused by prolonged paraquat (PQ) exposure. However, research on the precise mechanisms by which antioxidants influence the signaling pathways implicated in pulmonary fibrosis induced by paraquat is still insufficient. This research utilized a rat model of pulmonary fibrosis induced by PQ to examine the impacts of Silibinin (Sil) and cinnamic acid (CA) on pulmonary fibrosis, with a specific focus on pro-fibrotic signaling pathways and ROS-related autophagy. Lung injury induced by paraquat was demonstrated to be associated with oxidative stress and inflammation of the lungs, downregulated (miR-193a), and upregulated PI3K/AKT/mTOR signaling lung tissues. Expression levels of miR-193a were determined with quantitative real-time PCR, protein level of protein kinase B (Akt), and phosphoinositide 3-Kinase (PI3K) which were determined by western blot analysis. Hydroxyproline levels (HYP) and transforming growth factor-β1 (TGF-β1) were measured by ELISA, malondialdehyde (MDA), total antioxidant capacity (TAC), glutathione peroxidase (GSH), and catalase and were measured in lung tissue homogenates colorimetrically using spectrophotometer. Long-term exposure to paraquat resulted in decreased PI3K/AKT signaling, decreased cell autophagy, increased oxidative stress, and increased pulmonary fibrosis formation. Silibinin and cinnamic acid also decreased oxidative stress by increasing autophagy and miR-193a expression, which in turn decreased pulmonary fibrosis. These effects were associated by low TGF-β1. Silibinin and cinnamic acid inhibited PQ-induced PI3K/AKT by stimulating miR-193-a expression, thus attenuating PQ-induced pulmonary fibrosis.

**Supplementary Information:**

The online version contains supplementary material available at 10.1007/s00210-024-03511-y.

## Introduction

Paraquat (N,N′-dimethyl-4,4′-bipyridinium dichloride) is a quaternary nitrogen-containing compound and is a broad-spectrum herbicide that has increased agricultural production due to the variety of crops in which it is used. Other popular trade names for paraquat include methyl viologen, and paraquat dichloride (Dinis-Oliveira et al. 2008, Tsai 2013). In more than 120 countries around the world, over 100 crops are treated with paraquat, ranking it as the third most popular herbicide, due to its low cost (Dinis-Oliveira et al. 2008).

Paraquat (PQ) toxicity has resulted in many cases of human poisoning and deaths due to multiple organ failure, including the nervous system, heart, liver, and kidneys (Wright et al. 1997). Remarkably, paraquat accumulates mainly in lung tissue; as a result, PQ ingestion, whether accidental or intentional, may result in death due to progressive lung fibrosis and respiratory failure (Yan et al. 2017, Zeinvand-Lorestani et al. 2018).

Although the guidelines for the treatment of PQ poisoning patients have not yet been thoroughly established, current therapies include supportive care alone as well as combinations of hemodialysis, immunological modulation, antioxidant therapy, and hemoperfusion (Qian et al. 2014). Despite these treatments, the current mortality rate of PQ poisoning is still over 50% (Xu et al. 2015). Oxidative stress is a crucial molecular mechanism of paraquat-induced lung fibrosis; it often occurs in the lung after exposure to paraquat altering the usual balance between oxides and peroxides while increasing the quantities of reactive oxygen species (ROS) (Kinnula and Crapo 2003, Faner et al. 2012), such as superoxide anion (O^2−^), singlet oxygen (O), and hydroxyl and peroxyl radicals (Suntres 2002). ROS causes lung fibrosis by promoting lung cell death and reducing autophagy levels in alveolar epithelial cells (Jones 2006).

The most traditional and commonly used medicinal herb is Milk Thistle (*Silybum marianum*), which is used for its antioxidant properties (Negi et al. 2008). Silymarin is derived from the plant’s seeds and fruits and it was found to be a potent antioxidant (Comar and Kirby 2005). Silibinin (Sil) has also been shown to have powerful anti-proliferative effects against a variety of cancer cell lines (Gazak et al. 2007). Furthermore, a study of the cancer chemo-preventive and anticarcinogenic properties of silymarin found that the effects of silymarin are related to its major component Silibinin (Sil) (Hogan et al. 2007). Some studies have also proved that Silibinin has hepatoprotective effect against oxidative stress and ROS (Haddad et al. 2011).

Cinnamon is a plant with many uses as a herbal remedy. Its constituents include tannins, mucilage, resins, sugars, and essential oils. The most important of which is Cinnamaldehyde, which has anti-inflammatory, antibacterial, and antioxidant qualities (Barceloux 2009). Furthermore, cinnamic acid (CA), a primary active phenolic component in cinnamon, has a diversity of pharmacological activities, including antioxidant and antibacterial activity (Chen et al. 2011). CA has a strong antioxidant activity due to the existence of vinyl fragments in its structure. This property has sparked our interest in studying this natural compound as a potential treatment for pathological diseases caused by oxidative stress (Babaeenezhad et al. 2021).

The PI3K/AKT/mTOR signaling pathway is a key regulator of cell growth, proliferation, and survival. It is triggered by a variety of extracellular signals such as growth factors, cytokines, and hormones. The pathway consists of two main components: phosphatidylinositol 3-kinase (PI3K) and mammalian target of rapamycin (mTOR) (Shiau et al. 2022). The PI3K/Akt/mTOR signaling pathway is involved in the regulation of oxidative stress, which occurs when there is an imbalance between the cell’s ability to scavenge ROS and their production. Oxidative stress can be regulated via the PI3K/AKT/mTOR pathway, which modulates the expression of the antioxidant enzymes, catalase (CAT) and superoxide dismutase (SOD). Additionally, mTOR can regulate oxidative stress by modulating mitochondrial function and autophagy (Shiau et al. 2022).

The vital functions of microRNAs (miRNAs), an emerging class of gene expression regulators, in numerous biological processes, including cell proliferation, apoptosis, cell cycle progression and organ development, are well established (Muluhngwi and Klinge 2015). Also, miR193a was found to be methylated in patients with non-small cell lung cancer. An additional investigation revealed that miR193a-3p promoted apoptosis in lung cancer cells by adversely affecting ERBB4, a protein that is frequently aberrated in human lung cancer, thereby inhibiting cell invasion and proliferation (Liang et al. 2015).

The purpose of this research is to determine whether cinnamic acid and silibinin can protect against paraquat-induced pulmonary toxicity via the PI3K/Akt/mTOR pathway and miRNA 193-a signaling pathway.

## Materials and methods

### Materials

#### Animals

In this investigation, male adult Wistar albino rats weighing 200–250 g have been used. Clean plastic cages were used for keeping them. They were housed in an air-conditioned room maintained at 25 ± 2 °C with regular 12 h light/12 h dark cycle. Water and standard diet pellets were provided to them. To reduce physiological reactions to handling, rats were given a week to acclimatize.

#### Chemicals

Silibinin, cinnamic acid, and paraquat were purchased from Sigma-Aldrich Chemical Company (St. Louis, MO, USA). The remaining reagents and compounds were all of fine analytical grade.

### Methods

#### Experimental design

Six groups of male Wistar albino rats (200–250 gm) each consisted of 10 were randomly distributed.

- Group 1: normal control group (control), rats received saline 0.9% intraperitoneal (I.P.) and 0.5% carboxymethylcellulose (CMC) orally for seven consecutive days.
- Group 2: paraquat-toxified group (PQ), rats received 0.9% saline (I.P.) for 7 consecutive days, and then, on the 7^th^ day, they received a one single dose of paraquat dissolved in 0.9% saline (30 mg/kg, I.P.) (Ahmed et al. 2019).
- Group 3: (CA + PQ), rats received cinnamic acid (50 mg/kg, orally) dissolved in 0.5% CMC for 7 consecutive days followed by a one single dose of paraquat dissolved in 0.9% saline (30 mg/kg, I.P.) on the 7^th^ day.
- Group 4: (Sil + PQ), rats received Silibinin (200 mg/kg, orally) dissolved in 0.5% CMC for 7 consecutive days followed by a one single dose of paraquat dissolved in 0.9% saline (30 mg/kg, I.P.) on the 7^th^ day (Lu et al. 2009, Song et al. 2017).
- Group 5: (CA), rats received cinnamic acid (50 mg/kg, orally) dissolved in 0.5% CMC for 7 consecutive days (Abd El-Raouf et al. 2015).
- Group 6: (Sil), rats received Silibinin (200 mg/kg, orally) dissolved in 0.5% CMC for 7 consecutive days.

#### Collection of samples

All rats were sacrificed by being decapitated 48 hours after receiving the paraquat injection. The lungs were weighed after isolation. As soon as they were ready to be processed, all samples were kept at −80 °C.

#### Histopathological examination

Immediately after each animal was sacrificed, the upper left lung lobe was immersed in 10% neutral-buffered formalin. The lungs underwent a process of progressive dehydration, paraffin embedding, sectioning into 4μm, and subsequent staining with hematoxylin and eosin (H&E). An assessment was conducted on lung specimens to identify histological changes that are indicative of acute lung injury (ALI), including alveolar congestion, alveolar wall thickness and interstitial edema. The results were scored semi-quantitatively by METAVIR Scoring System on a scale of 0-3 for each item, where 0 = minimal damage, 1 = mild damage, 2 = moderate damage, and 3 = severe damage. A pathologist who performed the histopathological analysis was not informed about the experimental design of this study.

#### Preparation of lung tissue homogenates

Using a variable-speed homogenizer (tissue homogenizer yellow line D118 basic, Germany), the right lung tissues of the rats were immediately extracted and promptly homogenized in 0.09% biphosphate buffer (pH 7.4) at 4 °C. After centrifuging lung homogenates (Bench top cooling centrifuge model 2-16KL, Germany) for 15 min at 1000 g, 4 °C, supernatants were obtained to be used in assessing oxidative stress-related parameters and hydroxyproline.

#### Determination of hydroxyproline in lung tissue homogenate by ELISA

Through the measurement of hydroxyproline (HYP) levels, the collagen content of lung tissue can be determined. The presence of collagen deposition in the lungs is suggestive of lung fibrosis. The kits used were from CUSABIO, Houston, USA (Catalog No. CSB-E08838r). The competitive inhibition of enzyme immunoassay method is used in this assay. An antibody that is specific to HYP has been pre-coated onto the microtiter plate that is included in this kit. Standards or samples are put into the corresponding wells of the microtiter plate using horseradish peroxidase (HRP)-conjugated Hyp. The competitive inhibition reaction is launched between HRP-conjugated Hyp and Hyp in samples. The color that results from adding a substrate solution to the wells is inversely proportional to the quantity of Hyp present in the sample. The process of color development is stopped, and the color’s intensity is measured.

#### Determination of oxidative stress biomarkers in lung homogenate

The activity of malondialdehyde (MDA) and total antioxidant capacity (TAC) is determined by inhibiting chromogen reduction; this was accomplished using colorimetric assays obtained from Biodiagnostic, Giza, Egypt (Catalog No. MD 25 29 and Catalog No. TA 25 13). The MDA assay reagent operates by utilizing the reaction between MDA and thiobarbituric acid (TBA) in the sample to produce an MDA-TBA adduct. This adduct is subsequently quantified colorimetrically at an absorbance of 532 nm. On the other hand, the TAC assay relies on the conversion of Cu^2+^ to Cu^+^ via the action of small molecule antioxidants, such as glutathione (GSH). A colorimetric probe was utilized to chelate the reduced Cu^+^ ion, resulting in an absorbance peak at 570 nm O.D. that is directly proportional to the overall antioxidant capacity.

GSH levels in lung tissue homogenates were determined using reagents from Biodiagnostic (Catalog. No. GP 2524). The assay measures the activity of c-GPx in an indirect fashion. The reduction of an organic peroxide by c-GPx generates oxidized glutathione (GSSG), which is subsequently recycled back to its reduced state via the enzyme glutathione reductase (GR).

For determination of catalase (CAT), we performed a colorimetric assay using Biodiagnostic kit (Catalog No. CA 25-17). Its fundamental principle is that catalase must react with a predetermined amount of H_2_O_2_. The reaction stops precisely 1 min later when a catalase inhibitor is applied. When peroxidase (HRP) is present, the remainder of H_2_O_2_ undergoes a reaction with 3,5-dichloro-2-hydroxybenzene sulfonic acid (DHBS) and 4-aminophenazone (AAP). This reaction produces a chromophore whose wavelength of 510 nm is inversely proportional to the quantity of catalase present in the initial sample.

#### Determination of (transforming growth factor, *beta* 1) TGF-β1 using ELISA

This assay was performed using the FineTest biotechnology reagent, Wuhan, China (catalog No. ER1378). The sandwich enzyme-linked immune-sorbent assay technology formed the core of this reagent. The capture antibody was coated to 96-well plates via pre-coating. Additionally, detection antibodies were biotin-conjugated antibodies. Following the addition of the standards, test samples, and biotin-conjugated detection antibody to the wells, and to remove the mixture, a wash buffer was used. After adding HRP-Streptavidin, any unbound conjugates were removed by rinsing with wash buffer. By using TMB substrates, the HRP enzymatic reaction could be observed. TMB was removed from its blue form by HRP catalysis, which was reversed to yellow upon addition of an acidic solution to stop reaction. The density of yellow color is proportional to the quantity of the target sample that is captured on the plate. Using a microplate reader to measure the O.D., absorbance at 450 nm enables one to calculate the concentration of the target.

#### Determination of phosphatidyl inositol-3-kinase (PI3K) and protein kinase B (Akt) protein expression in the lung tissue of rats by western blot analysis

Lung tissue homogenate was centrifuged in accordance with a procedure already described. The supernatant was subsequently collected, and the concentration of total protein was determined using the Micro BCA Protein Assay Reagent.

Detection of PI3K was done by using Rabbit Polyclonal antibody to detect endogenous levels of PI3 Kinase p85/p55 protein only when phosphorylated at Tyr467/199, kit from Arigo Biolaboratories, Hsinchu City 300, Taiwan (Cat. No: ARG66216; Rabbit pAb). For determination of AKT, we used Akt1 (A-11) which is a mouse monoclonal antibody specific for an epitope mapping between amino acids 443-479 near the C-terminus of Akt1 of human origin, kit from Santa Cruz Biotechnology, Inc., Dallas, TX, USA (Cat. No: sc-377457).

Following electrophoresis on 8% polyacrylamide gel, protein samples were transferred to nitrocellulose membranes. Non-specific binding sites were blocked by incubating in TBST (0.05% Tween 20 in Tris buffered saline) and 7.5% (w/v) non-fat powdered milk for two hours at room temperature. After that, membranes were cleansed with TBST for 10 min. Subsequently, the primary specific antibodies targeting PI3K and Akt were diluted at a 1:1000 ratio and incubated at 4 °C overnight. Following membrane washing, the secondary antibody is horseradish peroxidase-conjugated anti-rabbit IgG antibody; it was applied at a 1:25000 dilution (Bio-Rad, USA) and incubated at room temperature for 1 h. This was subsequently followed by further rinsing. Using an enhanced chemiluminescence ECL Plus System (Amersham Biosciences, USA), immunocomplexes were observed.

Acquiring a high-quality digital image is the next step after western blot transfer. Next, the target protein bands, and the background are defined as regions of interest (ROI) using densitometry software. Band intensity is quantified using the Molecular Analyst Software (Bio-Rad, USA). For proper comparison, the results are adjusted to either a control protein or a background. Lastly, protein expression levels between samples can be analyzed using normalized densitometry measurements (Singh et al. 2021). The expression of PI3K and AKT proteins was quantified in relation to β-actin according to relative density.

#### Determination of miRNA 193-a using qRT-PCR

RNA extraction

Total RNA was extracted from the samples using the Direct-zol RNA Miniprep Plus Kit (Catalog No. R2072; ZYMO RESEARCH CORP.) The Direct-zol^TM^ RNA Miniprep Plus provides an efficient method for purifying up to 100 μg (per prep) of high-quality RNA directly from TRIzol samples. The TRI reagent sample was treated with ethanol, bound directly to the Zymo-Spin^TM^ column, and rinsed and the RNA was eluted. The RNA is of high quality and suitable for applications such as next-generation sequencing, reverse transcription-quantitative polymerase chain reaction (RT-qPCR), transcriptional profiling, and hybridization. The quantity and quality of RNA were assessed using a Beckman dual spectrophotometer. The ratio of absorbance at 260 and 280 nm was used to determine the purity of DNA and RNA. In general, a ratio of 1.8 is considered “pure” for DNA, while a ratio of 2.0 is considered “pure” for RNA.

### Quantitative real‐time polymerase chain reaction

One-step RT-PCR kit of the All-in-One™ miRNA qRT-PCR Detection Kit 2.0 (Gene Copeia TM, Rockville, USA) Cat. No. QP115 (20 RT and 200 qPCR reactions) and Cat. No. QP116 (60 RT and 600 qPCR reactions) were used for RNA reverse transcription followed by PCR in a single step (Table 1). The All-in-One™ RT-PCR System is designed for sensitive endpoint detection and analysis of RNA by RT-PCR. Using gene-specific primers, the formulation allows complementary DNA (cDNA) synthesis and PCR amplification to be performed in a single reaction tube. The All-in-One™ RT Master Mix enables highly efficient cDNA synthesis due to its high thermostability, processivity and ability to efficiently synthesize cDNA from a wide variety of RNA samples. The Master Mix is a 2X concentrated solution containing Moloney Murine Leukaemia Virus (M-MuLV) Reverse Transcriptase, Taq DNA Polymerases, SYBR Green Dye, ROX Dye, MgCl2 and buffer components at optimal concentrations. The M-MuLV enzyme has an optimal operating temperature and greater affinity for primer-template duplexes, allowing extremely rapid processing during the RT phase. Fluorescence is emitted when SYBR Green binds to double-stranded DNA. The prepared reaction mix samples were used in real-time PCR (Step One Applied Biosystem 7900, Foster City, CA, USA).

**Table 1:** Effect of cinnamic acid and Silibinin on paraquat-induced lung toxicity in rats by measuring miRNA-193a-3p using qRT-PCR

Primers	Forward	Reverse
miR-193a-3p	5′-GTT TGG TAG CTT ATC AGA CTG A-3′	5′-GTG CAG GGT CCG AGG T-3′
U6 (internal control)	5′-CTC GCT TCG GCA GCA CA-3′	5′-AAC GCT TCA CGA ATT TGC GT-3′

### Calculation of relative expression of the studied genes

The RT-PCR results were expressed as cycle threshold (Ct). The PCR data sheet includes the *C* values of the evaluated gene (miR-193-a) relative to the housekeeping gene (U6). A control sample should be used to assess the level of gene expression. The relative expression of each target gene is quantified and normalized to the housekeeping gene using the delta-delta Ct (ΔΔCt) calculation. The relative expression of each gene was calculated using 2-∆∆Ct.

## Statistical analysis

Data were analyzed statistically by SPSS (statistical package for social sciences), version 28, IBM software (NY, USA). The data were expressed as mean ± SD (standard deviation). SPSS was utilized to conduct one-way ANOVAs followed by Tukey post hoc tests for statistical comparisons between groups; *p*-values less than or equal to 0.001 considered to be significant.

## Results

### Histopathological examination of lung tissue

**Fig. 1:**
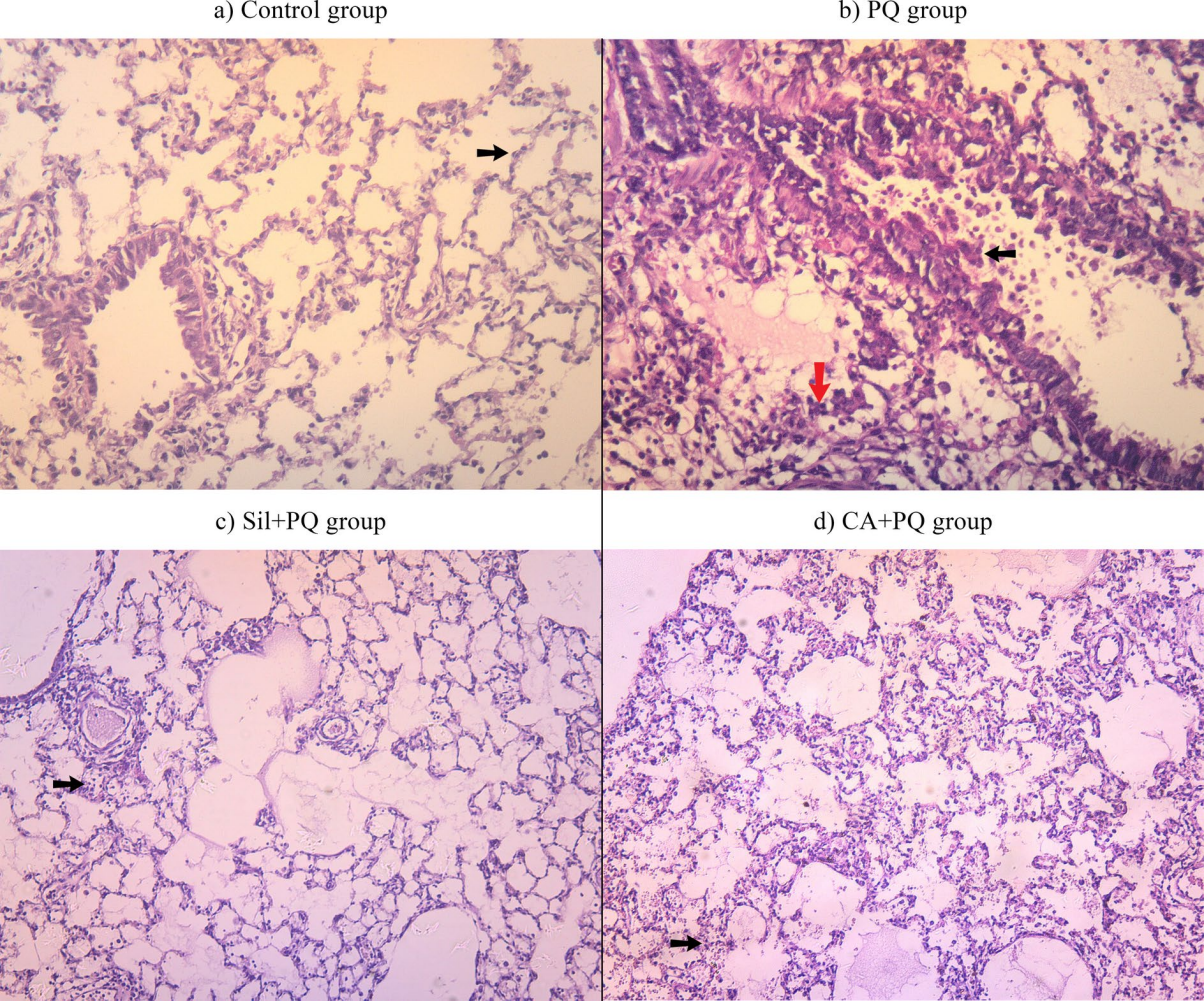
Representative images of hematoxylin and eosin (H&E)-stained lung tissues treated with paraquat (PQ), Silibinin with paraquat (Sil +PQ) and cinnamic with paraquat (CA+PQ). **a** Control (normal) group (H&E ×200 magnification). **b** PQ group, rats were administered PQ (30 mg/kg, intraperitoneal) single dose on the 7^th^ day (H&E ×200 magnification). **c** Sil + PQ group, pre-treated rats with Sil (200 mg/kg/day, orally for 7 days) then PQ (30 mg/kg) single dose, intraperitoneal on the 7^th^ day (H&E ×100 magnification). **d** CA + PQ group, pretreated rats with CA (50 mg/kg/day, orally for 7 days) then PQ (30 mg/kg) single dose intraperitoneal on the 7^th^ day (H&E ×100 magnification). Black arrows indicate the alveolar septa, red arrow indicates the peribronchial inflammations and epithelial desquamation

The histopathological examination for control group revealed severe fibrosis in the PQ-toxified lung tissue (diseased tissue) score = 3, in comparison to the normal lung tissue, score = 0. The alveolar spaces were patent and lined by normal pneumocytes in control group; however, the alveolar septa were markedly edematous, congested, and infiltrated by chronic inflammatory cells, and there were peribronchial inflammations and epithelial desquamation in the PQ group (Fig. 1a and b).

By pre-treating the rats with Sil and CA as protective antioxidants against PQ, the lung tissues revealed a potent prophylactic effect against PQ toxicity. The alveolar spaces were patent and the alveolar septa retained to the normal state with minimal residual inflammations in Sil + PQ group which indicates excellent response to toxicity, score of fibrosis = 1, whereas in CA + PQ group, the alveolar septa were mildly edematous, and congested and infiltrated by chronic inflammatory cells, which indicates moderate response to toxicity, score of fibrosis = 2, as shown in Fig. 1c and d.

### Oxidative stress markers in lung tissue homogenates in rats

**Fig. 2:**
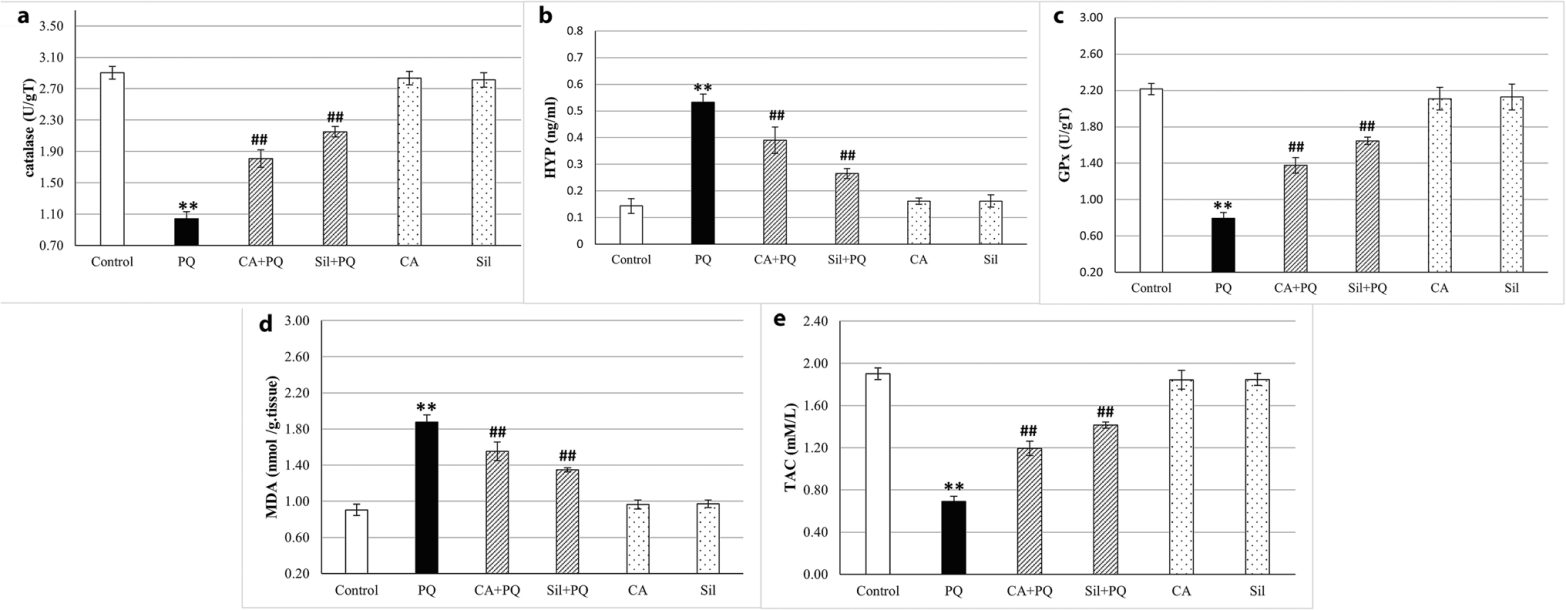
Graphical illustration of the effect of Silibinin and cinnamic acid on PQ-toxified rats on oxidative stress markers in the lung tissue of rats. **a** Comparison of catalase level (CAT) in lung tissue homogenate. **b** Comparison of hydroxyproline level (HYP) in lung tissue homogenate. **c** Comparison of glutathione peroxidase level (GPx) in lung tissue homogenate. **d** Comparison of malonaldehyde level (MDA) in lung tissue homogenate. **e** Comparison of total antioxidant capacity level (TAC) in lung tissue homogenate. CA (Cinnamic acid 50 mg/kg/day) and Sil (Silibinin 200 mg/kg/day) were given orally for 7 days prior to PQ (Paraquat 30 mg/kg, intraperitoneal, single dose on the 7^th^ day). Data were expressed as mean ± SD (*n* = 10). Data were analyzed statistically utilizing ANOVA and Tukey post hoc tests. ** differs significantly from the normal control group (*p <* 0.001), and ## differs significantly from the paraquat group (*p <* 0.001)

CAT level in the PQ group showed significant decrease by 64.13% in comparison to the normal control group (*p* < 0.001), on the other hand the CA and Sil administration to PQ-toxified rats significantly increased CAT level by 73.4% and 106.6% respectively at (*p* < 0.001) in comparison to PQ group (Fig. 2a, Table 2).

**Table 2:** The effect of Silibinin and cinnamic acid on paraquat-toxified rats p by measuring oxidative stress markers in the lung tissue of rats

Parameter	Control	PQ	CA + PQ	Sil + PQ	CA	Sil
CAT (U/gT)	2.9033±0.08	1.0414±0.09^**^	1.8067±0.11^##^	2.15±0.07^##^	2.835±0.08	2.812±0.09
TAC (mM/L)	1.9017±0.05	0.69±0.05^**^	1.1944±0.07^##^	1.415±0.03^##^	1.843±0.09	1.846±0.06
MDA (nmol/gT)	0.9033±0.06	1.8743±0.08^**^	1.5533±0.10 ^##^	1.3483±0.02^##^	0.963±0.05	0.97±0.04
GPx (U/gT)	2.215±0.06	0.7929±0.06^**^	1.3767±0.09^##^	1.645±0.04^##^	2.108±0.12	2.126±0.14
HYP (ng/ml)	0.143±0.03	0.532±0.03^**^	0.39±0.05^##^	0.265±0.02^##^	0.161±0.01	0.162±0.02

CA (Cinnamic acid 50 mg/kg/day) and Sil (Silibinin 200 mg/kg/day) were given orally for 7 days prior to PQ (Paraquat 30 mg/kg, intraperitoneal, single dose on the 7th day). Data were expressed as mean ± SD, (n=10). Data were analyzed statistically utilizing ANOVA and Tukey post hoc tests. ** differs significantly from the normal control group (*p* < 0.001), and ## differs significantly from the paraquat group (*p* < 0.001)

HYP level significantly increased in PQ group in comparison to normal control group by 271.7% (*p* < 0.001). On the other hand, the CA and Sil administration to paraquat-toxified rats significantly decreased HYP level in comparison to paraquat group by 26.77% (*p* < 0.001) and 50.19% (*p* < 0.001) respectively (Fig. 2b, Table 2).

GPx level in the PQ group is significantly decreased compared to the normal control group by 64.21% (*p* < 0.001); however, the CA and Sil administration to paraquat-toxified rats significantly increased GPx level compared to the PQ group by 73.7% (*p* < 0.001) and 107.5% (*p* < 0.001) respectively (Fig. 2c, Table 2).

MDA level in the PQ group is significantly increased by 107.3% compared to the normal control group (*p* < 0.001), while the CA and Sil administration to paraquat-toxified rats significantly decreased the MDA level by 17.11% and 28.1% respectively compared to the PQ group (*p* < 0.001) (Fig. 2d, Table 2).

TAC level in the PQ group showed significant decrease by 63.74% in comparison to the normal control group (*p* < 0.001); on the other hand, the CA and Sil administration to paraquat-toxified rats significantly increased the TAC level in comparison to the PQ group by 73.1% and 105.3% respectively (*p* < 0.001) (Fig. 2e, Table 2).

### TGF-β1 level using ELISA

**Fig. 3:**
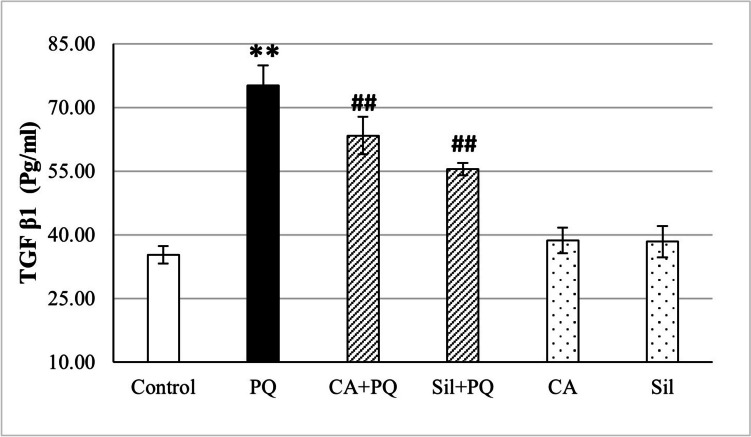
Effect of cinnamic acid and Silibinin on PQ-toxified rats by measuring TGF-β1 level in tissue homogenate using ELISA. CA (Cinnamic acid 50 mg/kg/day) and Sil (Silibinin 200 mg/kg/day) were given orally for 7 days prior to PQ (Paraquat 30 mg/kg, intraperitoneal, single dose on the 7^th^ day). Data were expressed as mean ± SD (*n* = 10). Data were analyzed statistically utilizing ANOVA and Tukey post hoc tests. ** differs significantly from the normal control group (*p <* 0.001), and ## differs significantly from the paraquat group (*p <* 0.001)

Paraquat significantly increased TGF-β1 level in the lung tissue homogenates of rats by 113.6% *p <* 0.001 in comparison to normal control group. However, CA and Sil administration to PQ-toxified rats significantly decreased the TGF-β1 level by 15.8% (*p <* 0.001) and 26.3% (*p <* 0.001) respectively, as we observe in Fig. 3.

### AKT and PI3K protein expression levels in lung tissue homogenates using western blot analysis

**Fig. 4:**
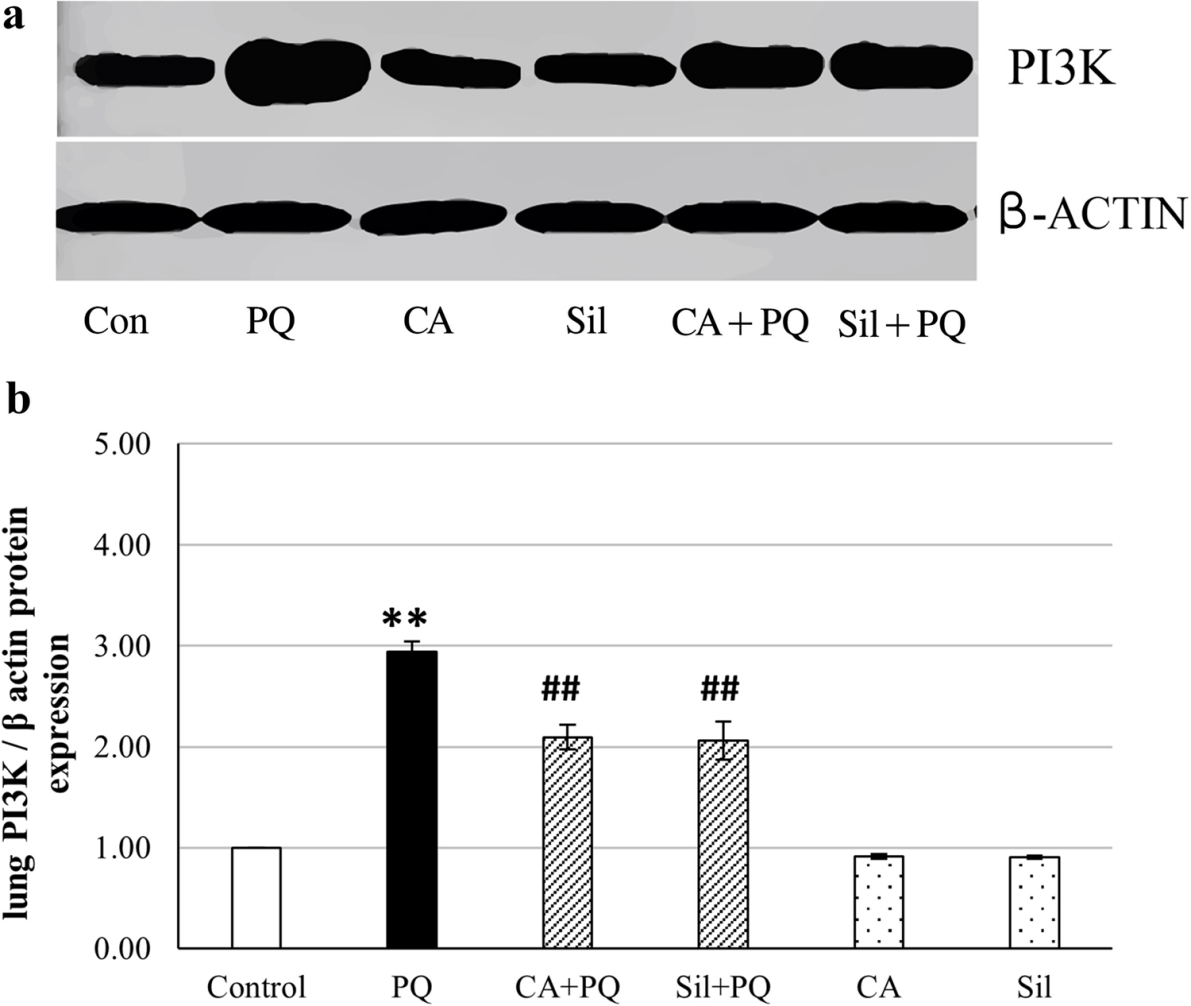
Effect of cinnamic acid and Silibinin on PQ-toxified rats by measuring phosphatidyl inositol-3-kinase (PI3K) protein expression level using western blot. **a** A representation of western blot of lung tissue PI3K protein expression. **b** Graphical illustration showing comparison of lung PI3K protein expression. CA (Cinnamic acid 50 mg/kg/day) and Sil (Silibinin 200 mg/kg/day) were given orally for 7 days prior to PQ (Paraquat 30 mg/kg, intraperitoneal, single dose on the 7^th^ day). Data were expressed as mean ± SD (*n* = 10). Data were analyzed statistically utilizing ANOVA and Tukey post hoc tests. ** differs significantly from the normal control group (*p <* 0.001), and ## differs significantly from the paraquat group (*p <* 0.001)

**Fig. 5:**
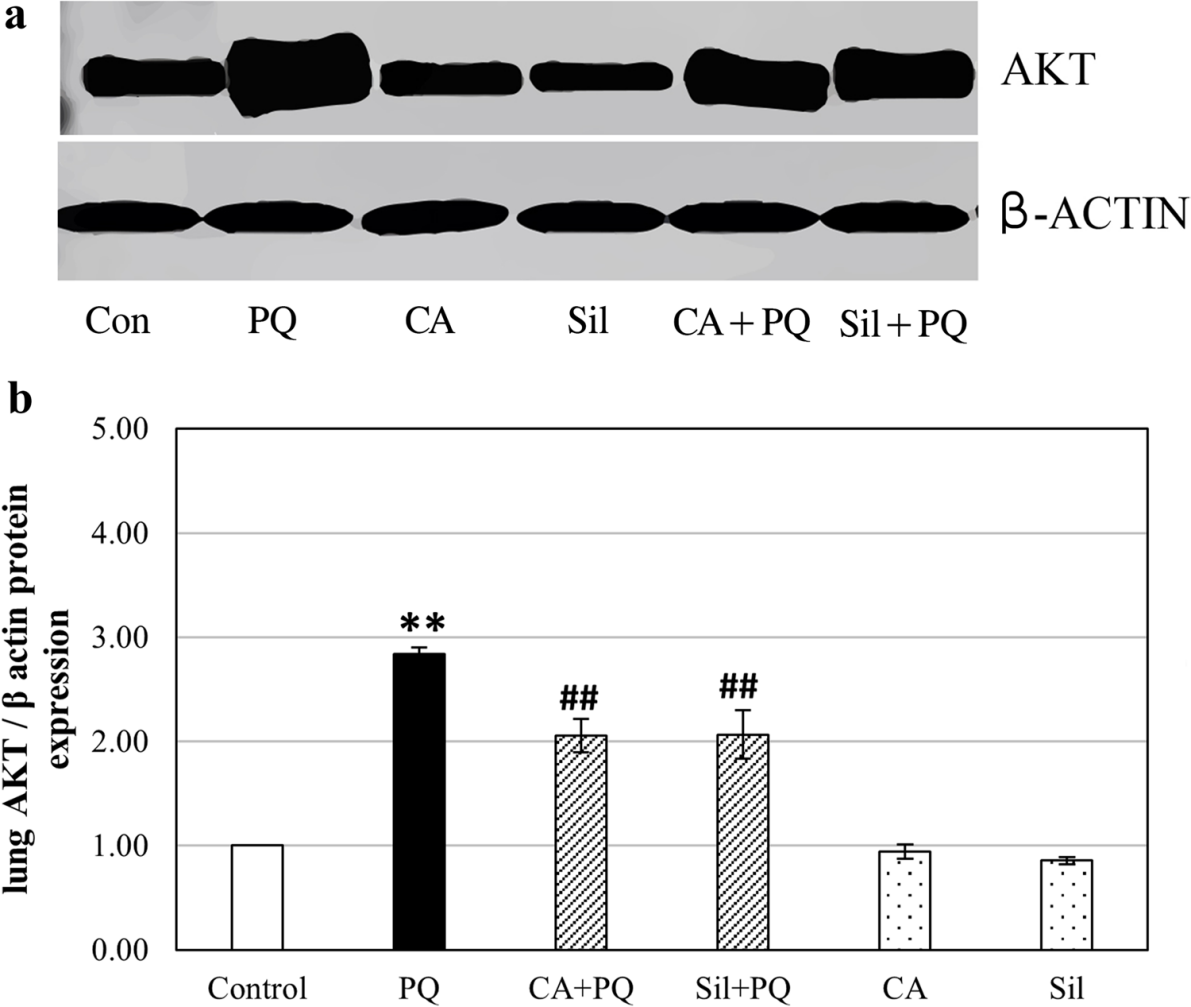
Effect of cinnamic acid and Silibinin on PQ-toxified rats by measuring protein kinase B (AKT) protein expression level in lung tissues of rats using western blot. **a** A representation of western blot of lung tissue AKT protein expression. **b** Graphical illustration showing comparison of lung AKT protein expression level. CA (Cinnamic acid 50 mg/kg/day) and Sil (Silibinin 200 mg/kg/day) were given orally for 7 days prior to PQ (Paraquat 30 mg/kg, intraperitoneal, single dose on the 7^th^ day). Data were expressed as mean ± SD (*n* = 10). Data were analyzed statistically utilizing ANOVA and Tukey post hoc tests. ** differs significantly from the normal control group (*p <* 0.001), and ## differs significantly from the paraquat group (*p <* 0.001)

The expression levels of the PI3K and AKT proteins in the lung tissue of rats that administered Paraquat increased significantly by 158.7% (*p* < 0.001) and 193% (*p* < 0.001) respectively, in comparison to the normal control group. On the other hand, the administration of cinnamic acid and Silibinin to PQ-toxified rats showed a significant decrease in PI3K protein expression level in the lung tissues of rats by 22.4% (*p* < 0.001) and 22% (*p* < 0.001) respectively, compared to PQ group, as well as a significant decrease in AKT protein expression level in the lung tissues of rats by 26% (*p* < 0.001) and 27.6% (*p* < 0.001) respectively compared to PQ group, as illustrated in Fig. 4 and Fig. 5.

### The expression level of miRNA-193a-3p using qRT-PCR

**Fig. 6:**
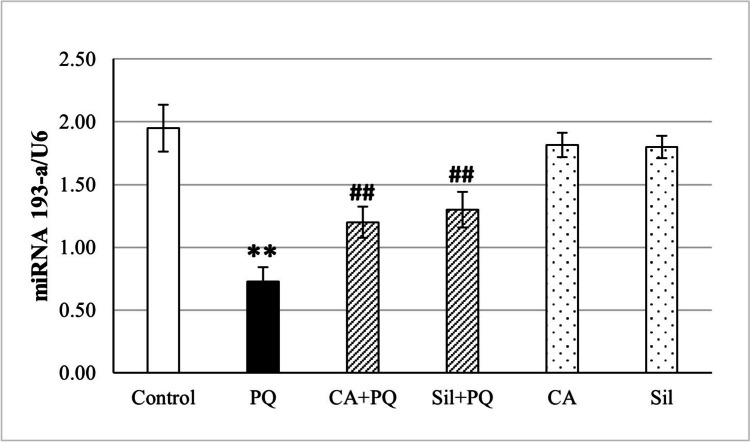
Effect of cinnamic acid and Silibinin on PQ-toxified rats by measuring miRNA 193-a gene expression by RT-PCR. CA (Cinnamic acid 50 mg/kg/day) and Sil (Silibinin 200 mg/kg/day) were given orally for 7 days prior to PQ (Paraquat 30 mg/kg, intraperitoneal, single dose on the 7^th^ day). Data were expressed as mean ± SD (*n* = 10). Data were analyzed statistically utilizing ANOVA and Tukey post hoc tests. ** differs significantly from the normal control group (*p <* 0.001), and ## differs significantly from the paraquat group (*p <* 0.001)

We explored the effects of Silibinin and cinnamic acid on miRNA 193-a expression levels in fibrotic tissues of lung obtained from rats receiving one single dose of PQ (30 mg/kg). MiRNA-193a expression levels in lung tissues were assessed with qRT-PCR. MiR-193a expression levels were decreased in the lungs during paraquat-induced lung fibrosis in comparison to the normal control group by 62% (*p* < 0.001). Conversely, miR-193a expression was significantly increased in cinnamic acid and Silibinin-treated rats in comparison to the Paraquat group by 64.7% (*p* < 0.001) and 78.5% (*p* <0.001) respectively (Fig. 6).

### Correlation between miRNA 193a and other lung parameters

**Fig. 7:**
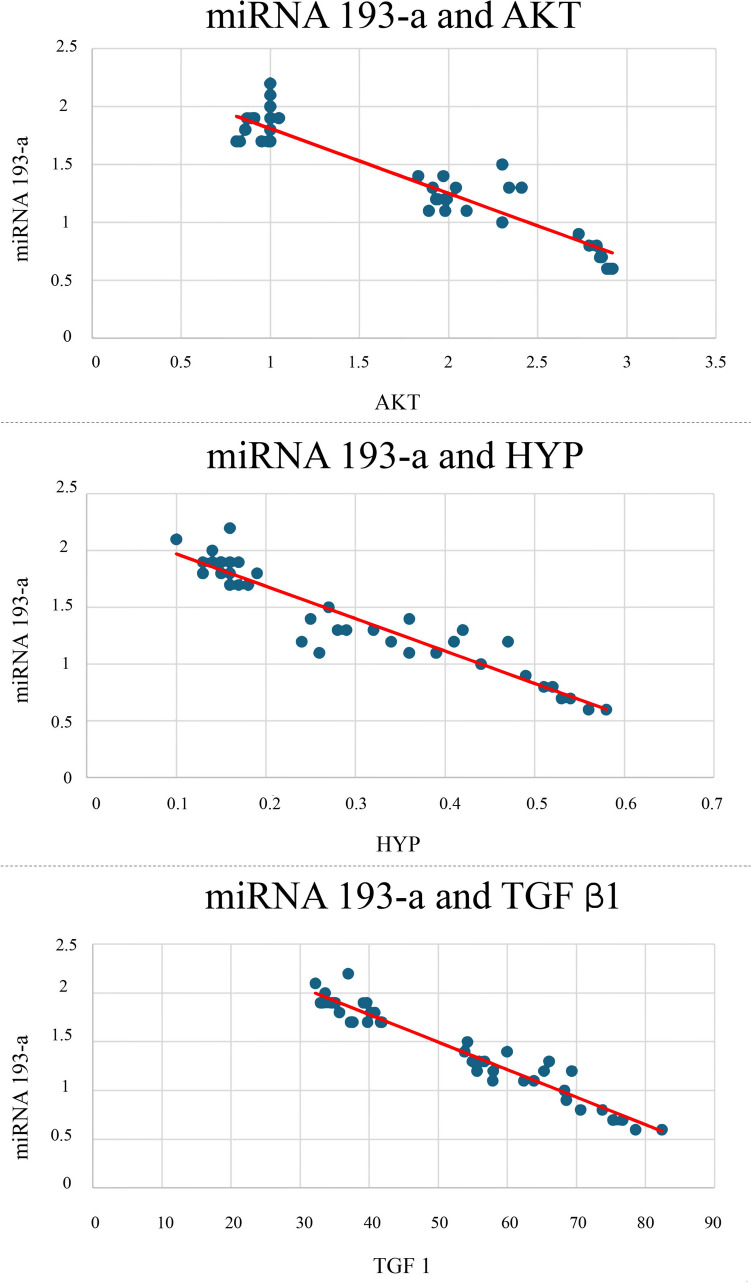
A scattered chart showing **a** the correlation between miRNA 193-a and AKT ( protein kinase B), *r = −*0.94 at *p <* 0.001. **b** The correlation between miRNA 193-a and HYP (hydroxyproline), *r = −*0.94 at *p <* 0.001. **c** The correlation between miRNA 193-a and TGF β1 (transforming growth factor β1), *r = −*0.96 *p <* 0.001

As illustrated in Fig. 7, a statistically strong inverse correlation was observed between miRNA 193-a on one hand, and AKT, HYP, and TGF β1 on the other hand, where *r* = −0.94, *r* = −0.94, and *r* = −0.96 respectively, all at *p* < 0.001.

### Graphical Abstract

**Fig. 8:**
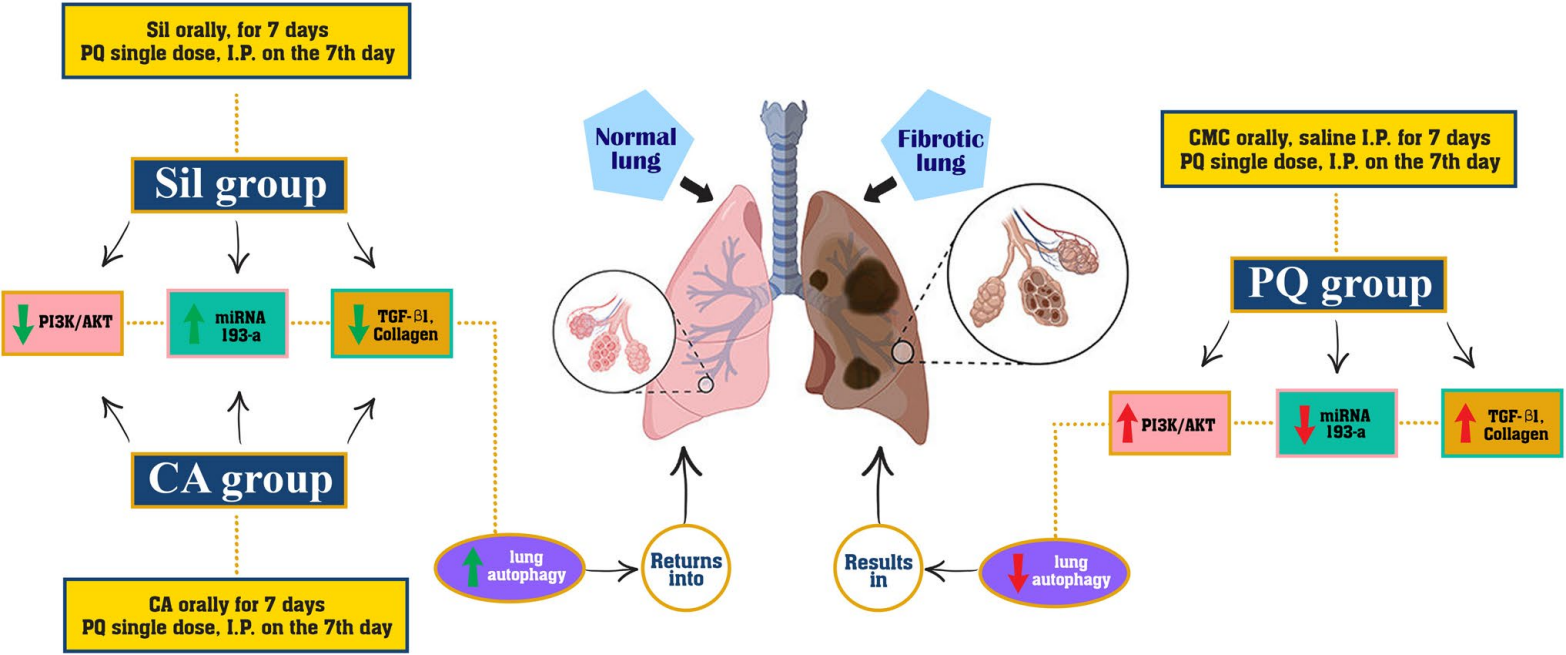
Illustrated scheme for the mechanism of each of paraquat, cinnamic acid, and Silibinin on lung toxicity and fibrosis. CA (Cinnamic acid 50 mg/kg/day) and Sil (Silibinin 200 mg/kg/day) were given orally for 7 days prior to PQ (Paraquat 30 mg/kg, intraperitoneal (I.P), single dose on the 7^th^ day)

This graph presents and illustrates our study in brief, where it shows the effect of each of paraquat, Silibinin, and cinnamic acid on lung tissue of rats and pulmonary fibrosis (Fig. 8). Also, it shows the mechanism of PI3K/AKT pathway and its relationship with miRNA 193-a, TGF-β1, and collagen deposition in the lungs.

## Discussion

Paraquat has the third-highest global pesticide usage rate. One of the many negative outcomes of paraquat poisoning is the development of Parkinson’s disease and severe lung damage. PQ’s capacity to induce substantial lung damage in both experimental animals and humans has led to its identification as a major health concern. As a pulmonary toxicant, PQ’s toxicity has been the subject of substantial research (Tyagi and Singh 2020).

The prophylactic effects of cinnamic acid and Silibinin against paraquat-induced lung toxicity may be attributed to their antioxidant and anti-inflammatory properties. These compounds have been reported to scavenge free radicals, reduce oxidative stress, and inhibit the production of pro-inflammatory cytokines. Additionally, cinnamic acid and Silibinin may modulate various signaling pathways involved in oxidative stress and inflammation, such as inhibition of the PI3K/AKT pathway that enhance the expression of antioxidant enzymes and promote cellular defense against oxidative damage. Furthermore, cinnamic acid and Silibinin have been shown to possess anti-fibrotic properties, which may contribute to their protective effects against lung toxicity. These compounds can inhibit the activation of fibroblasts and the deposition of extracellular matrix components, thereby preventing tissue fibrosis (Koczurkiewicz-Adamczyk et al. 2021, Ray et al. 2024).

Prior research has established that reactive oxygen species, namely hydrogen peroxide and superoxide radicals, are produced when the enzyme NADPH-Cyt-p-450 reductase metabolizes paraquat (Yumino et al. 2002). The accumulation of superoxide radicals has the potential to induce lipid peroxidation, reduce the overall antioxidant capacity, and promote cytokine formation in lung tissues (Tyagi and Singh 2020). Additionally, the elevated lung hydroxyproline levels in the current study may serve as an early indicator of fibrosis, as hydroxyproline is a major component of collagen (Huang et al. 2021).

The present study showed that when lung tissue homogenates were pre-treated with cinnamic acid and silibinin before PQ intoxication, the levels of the antioxidant markers CAT, TAC, and GPx were significantly higher than in the PQ group. In contrast, the levels of the oxidative marker MDA and the profibrotic marker HYP were significantly lower. This came in accordance with prior research that reported that CA is effective against cisplatin toxicity by reducing some inflammatory markers (El-Sayed et al. 2013). Also, Lu et al. (2009) reported that Silibinin reduces peroxidation of lipids, in hepatocyte microsomes and isolated hepatocytes, suggesting that it may provide protection against oxidative stress.

Among all cytokines that have been examined thus far, the TGF-b family of proteins exhibits the most potent stimulatory effect on extracellular matrix deposition. Studies conducted in vitro have demonstrated that TGF-b1, which is secreted as a latent precursor, stimulates the expression of the procollagen gene in fibroblasts and their protein synthesis (Xiangdong et al. 2011). TGF-β1 increased alveolar permeability of the epithelial cells in vitro through a mechanism involving intracellular glutathione depletion. Anti-TGF-β1 antibodies prevented lung injury in hemorrhaging rodents, according to another study (Shenkar and Coulson 1994).

Our results revealed that PQ induced TGF-β-1 activity, but pre-treatment with cinnamic acid and silibinin reduced its levels in PQ-intoxicated lungs. This comes in accordance with the previous studies done by Barceloux (2009) that proved that cinnamon extracts has potent antioxidant activity and also reduced the levels of TGF-β1. Furthermore, a previous study proved that Silibinin promoted valsartan’s anti-fibrotic effect by inhibiting the TGF-β1 signaling pathway (Liu et al. 2020). Presently, among all cytokines studied, the growth factor β family of proteins has shown the most significant effect on the deposition of extracellular matrix. TGF-β1, which is secreted in vitro as a latent precursor, stimulates the expression of fibroblast procollagen genes and the synthesis of collagen protein (Xiangdong et al. 2011). TGF-β1 increased alveolar permeability of the epithelial cells in vitro through a mechanism involving intracellular glutathione depletion. Anti-TGF-β1 antibodies prevented lung injury in hemorrhaging rodents, according to another study (Shenkar and Coulson 1994).

Results of our studies showed that Silibinin and cinnamic acid inhibited AKT and PI3K in PQ-intoxified rats. Akt and PI3K are phosphorylated by growth factors and cytokines in response to physiological stimuli, which stimulate tuberous sclerosis 1 proteins, which subsequently stimulates mTOR. And as previously described, paraquat contributes to the pathogenesis of pulmonary fibrosis by activating the PI3K/Akt pathway in alveolar epithelial cells and inhibiting autophagy activity (Jiang et al. 2021, Shi et al. 2022). Silibinin has previously been shown to inactivate the PI3K/Akt pathway in kidney cancer cells (Yassin et al. 2021); moreover, cinnamic acid also inhibited the propagation of gastric cancer cells by downregulating the PI3K/Akt pathway. And as confirmed from previous studies done by Li and Hu (2020), the proteins PI3K/ AKT are recognized for their involvement in the processes of wound healing. Also, Kim et al. (2017) showed that stimulation of the PI3K/AKT signaling pathway is recognized as the fundamental factor in keloid pathogenesis by inducing collagen synthesis.

Regarding the present study, the rats treated only with PQ have lower expression levels of miRNA 193-a than those pre-treated with cinnamic acid and Silibinin. Prior researches proved that Paraquat has impact on the miRNA 193a via down regulation its expression levels and thus inducing oxidative stress in lung cells (Liu et al. 2019). miR-193a inhibited lung cancer cell proliferation and invasion while promoting apoptosis (Liang et al. 2015). Studies done by Fan et al. 2017. and Khordadmehr et al. (2019) showed that patients diagnosed with non-small cell lung cancer exhibited a distinct pattern of miR-193a methylation. It was shown that miR-193a-3p may modulate the signaling of the PI3K/Akt pathway by targeting KRAS and may function as a tumor suppressor in NSCLC tissues by analyzing their expression patterns.

## Conclusion

Our study illustrated that paraquat could induce pulmonary toxicity and fibrosis by enhancement of oxidative stress and collagen deposition in lung tissues and consequently increasing level of TGF-β1, also affecting pathway of PI3K/AKT by inducing it and inhibiting the signaling of miRNA 193-a in lung tissues. Cinnamic acid and Silibinin on the other hand offered a new prophylactic effect against PQ toxicity by downregulation of PI3K/AKT pathway and increasing the expression level of miRNA 193-a in the lung tissues. According to our study, we found that Silibinin has a more potent antioxidant effect than cinnamic acid in most of the oxidative parameters and better prophylactic effect considering the histopathology images taken for the lung tissues.

More studies are recommended to test if there is a synergistic effect from combining cinnamic acid and Silibinin as protective agents against lung toxicity. Investigation of the pulmonary protective effects of cinnamic acid and Silibinin in patients intoxicated with paraquat is recommended through clinical trials.

## Supplementary Information

ESM 1 (PNG 705 kb)

High resolution Image (TIF 2083 kb)

ESM 2 (DOCX 19 kb)

## Data Availability

No datasets were generated or analyzed during the current study.
