# Supplementary figures and images for: Protective effects of Silibinin and cinnamic acid against paraquat-induced lung toxicity in rats: impact on oxidative stress, PI3K/AKT pathway, and miR-193a signaling
Source: Naunyn Schmiedebergs Arch Pharmacol. 2024 Oct 25;398(4):4291–303. doi: 10.1007/s00210-024-03511-y (PMC11978700; doi:10.1007/s00210-024-03511-y)

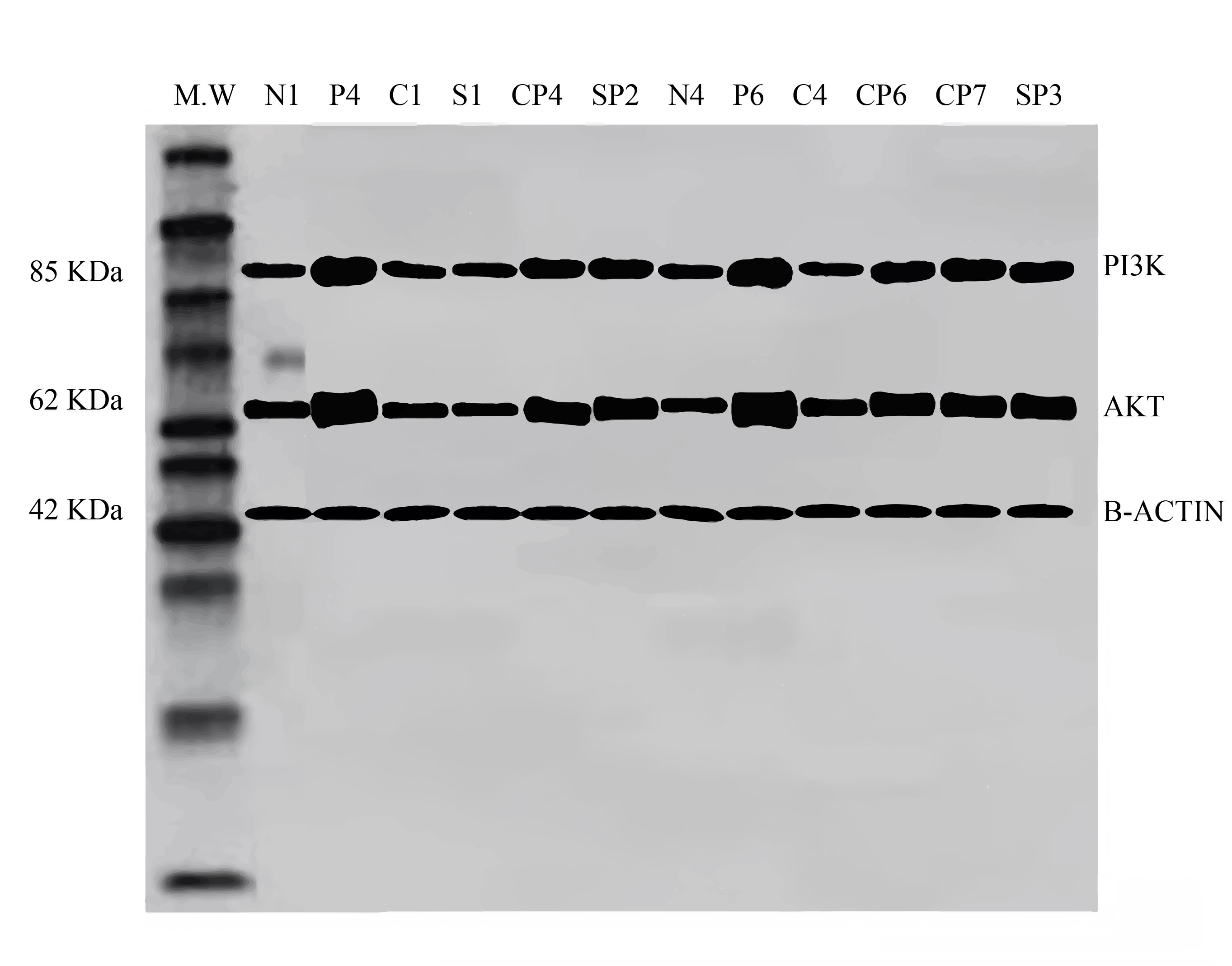

Supplement: Supplementary file 1 — (PNG 705 kb) [file 210_2024_3511_Fig9_ESM.png]
