# Supplementary material for: Protective effects of Silibinin and cinnamic acid against paraquat-induced lung toxicity in rats: impact on oxidative stress, PI3K/AKT pathway, and miR-193a signaling
Source: Naunyn Schmiedebergs Arch Pharmacol. 2024 Oct 25;398(4):4291–303. doi: 10.1007/s00210-024-03511-y (PMC11978700; doi:10.1007/s00210-024-03511-y)
Supplement: Supplementary file 3 — (DOCX 19 kb) [file 210_2024_3511_MOESM2_ESM.docx]

**Survival rate**

Survival rate of rats is done after induction of Paraquat toxicity to groups (PQ) , (CA+PQ) and ( Sil + PQ) till the day of their sacrifice.

As we see survival rate in CA+PQ and Sil+PQ groups was better than PQ group.

Where CA (Cinnamic acid 50 mg/kg/day) and Sil (Silibinin 200 mg/kg/day) were given orally for 7 days prior to PQ (Paraquat 30 mg/kg, intraperitoneal, single dose on the 7th day). Rats were sacrificed after 48 hours of PQ induction.

| **Number of survived rats** | **PQ (15)** | **CA+PQ (15)** | **Sil+PQ (15)** |
| --- | --- | --- | --- |
| **Day of PQ induction** | 15 | 15 | 15 |
| **After 24 hr** | 12 | 14 | 14 |
| **After 48 hr** | 11 | 13 | 14 |
